# Effect of Trainee Level on Surgical Time and Postoperative Complications of Anterior Cruciate Ligament Reconstruction 

**DOI:** 10.5435/JAAOSGlobal-D-23-00037

**Published:** 2023-05-02

**Authors:** Priyanka Parameswaran, Yash Tarkunde, J. Sam Broughton, Michael G. Rizzo, Jake Goldfarb, Robert H. Brophy

**Affiliations:** From the Department of Orthopaedic Surgery, Washington University School of Medicine, St. Louis, MO (Ms. Parameswaran, Mr. Tarkunde, Mr. Broughton, and Dr. Brophy); Williams College, Williamstown, MA (Mr. Goldfarb); and the Department of Orthopaedic Surgery, University of Miami Miller School of Medicine, Miami, FL (Dr. Rizzo).

## Abstract

The objective of this study was to investigate the association between trainee level and surgical time and postoperative complications of anterior cruciate ligament reconstruction (ACLR).

**Methods:**

A retrospective chart review of patients who underwent ACLR at an academic orthopaedic ambulatory surgery center collected demographic and clinical information, including the number of trainees present and trainee level. Unadjusted and adjusted regression analyses assessed the association between trainee number and level with surgical time (time from skin incision to closure) and postoperative complications.

**Results:**

Of 799 patients in this study operated on by one of five academic sports surgeons, 87% had at least one trainee involved. The average surgical time overall was 93 ± 21 minutes and by trainee level was 99.7 (junior resident), 88.5 (senior residents), 96.6 (fellows), and 95.6 (no trainees). Trainee level was significantly associated with surgical time (*P* = 0.0008), with increased surgical time in cases involving fellows (0.0011). Fifteen complications (1.9%) were observed within 90 days of surgery. No notable risk factors of postoperative complications were identified.

**Conclusion:**

Resident trainee level does not have a notable effect on surgical time or postoperative complications for ACLR at an ambulatory surgery center, although cases involving fellows had longer surgical times. Trainee level was not associated with risk of postoperative complications.

Resident and fellow training is a fundamental part of medical education,^1^ but its effect on patient care and efficiency is increasingly under scrutiny.^2^ Studies have shown that teaching residents increases surgical time in certain obstetrics/gynecology,^3^ general surgery,^4^ and otolaryngology procedures.^5^ Investigations of orthopaedic procedures have focused primarily on total joint arthroplasty, with evidence showing variable effects on surgical time and no change in postoperative complications or patient-reported outcome measures.^6,7^ There is little research to date on the effect that trainee learning curves might have in different surgical settings.

As expectations for surgeon efficiency increase, especially in the ambulatory surgery center (ASC) environment,^8^ it is important to understand how the learning curve of trainees effects surgical intervention and patient outcomes. The purpose of this study was to test the hypothesis that trainee level is associated with surgical time and postoperative complications of anterior cruciate ligament (ACL) reconstruction, one of the most common orthopaedic, ASC procedures. We hypothesize that there will be notable differences in mean surgical time across trainee levels.

## Methods

### Study Participants

With approval from the institutional review board, a retrospective chart review of patients who underwent ACL reconstruction over a 3-year period (June 1, 2015, through June 1, 2018) at a freestanding, academic orthopaedic ASC, by one of five members of an academic department of orthopaedic surgery with fellowship training in sports medicine was conducted. Patients undergoing ACL reconstruction with concomitant meniscal surgery, cartilage débridement/chondroplasty, and loose body removal were included, but those undergoing other ligament surgery, osteotomy, or a articular cartilage restoration procedure were excluded. Because this study was designed to focus on surgical time and short-term complications, patients had to have documented follow-up in the chart of at least 90 days. Of the patients initially eligible for inclusion, 83.7% (878/1049) had at least 90 days of follow-up. A total of 799 patients met criteria and were included in the cohort. Participants were grouped based on the type of surgery: isolated ACL reconstruction; ACL reconstruction and meniscal repair with or without additional procedures such as meniscal débridement, chondroplasty, or loose body removal; ACL reconstruction and meniscal débridement with or without additional procedures such as chondroplasty or loose body removal; and ACL reconstruction with chondroplasty or loose body removal. Revision ACL surgeries were coded with a separate variable indicating that the procedure was a revision.

### Data Acquisition

Data were collected through automated extraction of the electronic medical record, in collaboration with the university perioperative systems team and Clinical Investigation Data Exploration Repository, as described in a previous study.^9^ All surgeries were conducted at one of our sites, an outpatient surgery center. Queries were run on all ACL reconstructions during the study period, and a chart review was used to check for database accuracy and to finalize missing data. Patient and surgeon data, including attending surgeon, patient age, anesthesia type, length of surgery, and surgical time, were automatically extracted. Demographic information, including age, sex, and body mass index (BMI), were collected manually from the electronic medical record. Information about postoperative complications, including infection, DVT/PE, wound dehiscence/hematoma evacuation, arthrofibrosis, and graft failure, was collected from Epic medical records (Verona). Records were reviewed through the date of last follow-up for each patient, and the length of follow-up from the date of surgery was recorded.

### Trainee Level

We reviewed the surgical report for each patient and collected the total number of trainees present (including medical students, residents, and fellows). The names of all trainees present were also obtained from the surgical note; trainee names were subsequently converted to postgraduate years (PGYs) by consulting the university's residency class rosters. Trainee level was categorized as no trainee, junior residents (PGY1-3), senior residents (PGY4-5), or fellow. In cases where multiple trainees were present, trainee level was assigned according to the most senior trainee in the surgery. The database did not include data on whether advanced practice providers were involved in any of the surgeries because they are typically not used in this setting. Procedures with only medical students were classified as no trainee.

### Surgical Outcomes

Outcomes of interest included surgical time and postoperative complications. Surgical time was defined as the time from skin incision to closure and was documented in the database. Postoperative complications, including infection, DVT/PE, wound dehiscence/hematoma evacuation, arthrofibrosis requiring surgical débridement, and graft failure, were obtained from a manual chart review. Complications were categorized into three time frames: 0 to 30 days, 31 to 90 days, or > 90 days postoperatively. The maximal length of follow-up for the cohort was up to 4 years after the initial surgery.

### Statistical Analysis

We conducted unadjusted and adjusted regression analyses to investigate the association of trainee number and trainee level with surgical time and postoperative complications. Descriptive statistics were used for demographic data, and one-way analysis of variance (continuous variables) and the chi square or Fisher exact test (categorical variables) were used to assess any patient demographic differences between trainee levels. Unadjusted bivariable analysis was used to compare surgical times across trainee levels.

### Power Analysis

A power analysis for the multivariable linear regression model with an alpha value of 0.05 and beta value of 0.20 resulted in a minimum sample size of 489 to detect a difference in mean surgical time. The power analysis for the logistic regression model with an alpha value of 0.05 and beta value of 0.20 resulted in a minimum sample size of 329 to detect a difference.

### Multivariable Analysis

Linear regression analysis was conducted with 10 independent variables to determine the significance and effect size of each variable. These variables included age, sex (male and female), BMI, history of diabetes, smoking history, procedure category, whether the procedure was a revision surgery, attending surgeon, number of trainees, and trainee level. Age, BMI, and total number of trainees were continuous variables; all other variables were categorical. Our outcome of interest was surgical time. All factors included in analysis were identified a priori. General linear models with parameter estimates and effect size (partial eta squared) were created for surgical time.

Logistic regression analysis was conducted to determine which variables were markedly associated with the presence or absence of a complication. The same 10 independent variables described earlier were included in the logistic regression models. Models were created for all complications, and all complications that occurred less than 90 days postoperatively. Logistic models were also created for each individual complication type: infection, DVT/PE, wound dehiscence/hematoma evacuation, arthrofibrosis, and graft failure. A significance level of 0.1 was required for model entry, and a significance of 0.05 was needed to remain in the model. The Firth penalized score procedure was used to control for quasi-complete separation in rare event analysis for all logistic models.^9-11^ Penalized odds ratios and 95% confidence intervals are reported. All statistical tests were conducted with a significance threshold of α = 0.05, and effect sizes were estimated with partial eta squared. Analysis was conducted with SAS (Cary).

### Competing Interests

There were no financial, institutional, or general competing interests.

## Results

There were 799 patients included in this study; most of these patients (87%) had at least one trainee involved in their surgery. The mean age for the cohort was 25.8 years (SD = 12.0), and 51.2% of patients were men. The mean BMI was 25.7 (SD = 5.0). The mean length of follow-up was 238 (SD = 123, range: 90 to 1072) days after surgery. Of surgeries that included a trainee, 78.9% had one trainee, 8.7% had two trainees, and 1% had three trainees. No significant patient demographic differences were observed across trainee levels (Table 1); however, trainee level was associated with surgical time (*P* < 0.0001), attending surgeon (*P* < 0.0001), and the total number of trainees involved in the case (*P* < 0.0001).

**Table 1:** Participant Demographics

	No Trainee	Junior Resident	Senior Resident	Fellow	*P* value
**Total no.**	107	116	418	158	
**Age (yrs) (mean [SD])**	24.3 (10.7)	26.8 (12.1)	25.9 (12.2)	25.9 (12.2)	0.4657
**Sex (no. [%])**					0.8076
Men	54 (50.5)	60 (51.7)	209 (50)	86 (54.4)	—
Women	53 (49.5)	56 (48.3)	209 (50)	72 (45.6)	—
**BMI**	26.1 (6.2)	25.7 (4.3)	25.4 (4.6)	26.2 (5.4)	0.2679
**Total # of trainees (no. [%])**					<0.0001
1	0 (0)	0 (0)	380 (90.9)	131 (82.9)	—
2	0 (0)	104 *(89.7)*	34 (8.1)	23 (14.6)	—
3	0 (0)	12 (10.3)	4 (1.0)	4 (2.5)	—
**Attending (no. [%])**					<0.0001
Surgeon 1	13 (12.1)	15 (12.9)	4 (1.0)	2 (1.3)	—
Surgeon 2	52 (48.6)	28 (24.1)	82 (19.6)	48 (30.4)	—
Surgeon 3	20 (18.7)	5 (4.3)	194 (46.4)	62 (39.2)	—
Surgeon 4	15 (14.0)	68 (58.6)	9 (2.1)	25 (15.8)	—
Surgeon 5	7 (6.5)	0 (0)	129 (30.9)	21 (13.3)	—
**Diabetes (no. [%])**					0.059
Yes	3 (2.8)	0 (0)	2 (0.5)	3 (1.9)	—
No	104 (97.2)	116 (100)	415 (99.5)	155 (98.1)	
**Asthma (no. [%])**					0.2484
Yes	16 (15.1)	18 (15.6)	43 (10.4)	20 (12.7)	—
No	90 (84.9)	97 (84.4)	372 (89.6)	138 (87.3)	—
**RA (no. [%])**					0.559
Yes	0 (0)	1 (1.0)	1 (0.2)	1 (0.3)	—
No	106 (100)	114 (99)	416 (99.8)	157 (99.3)	—
**Smoking status (no. [%])**					0.5578
Current	2 (1.9)	5 (4.3)	17 (4.1)	6 (3.8)	—
Former	6 (5.7)	2 (1.7)	8 (1.9)	3 (1.9)	—
Never	98 (92.4)	108 (93.9)	388 (93.9)	148 (94.3)	—
**Surgical time (min) (mean [SD])**	95.6 (28.5)	99.7 (24.4)	88.5 (16.8)	96.6 (17.5)	<0.0001

BMI = body mass index

The mean surgical time was 93 minutes (SD = 21 minutes). Eighty-five complications (10.6%) were found in the cohort (Table 2), 15 (1.9%) of which occurred less than 90 days after surgery. Longer term complications included arthrofibrosis and graft failure, with some complications occurring years after the initial surgery.

**Table 2:** Postoperative Complications

Complication	Total	1-30 days	31-90 days	90+ days
Infection (including cellulitis and stitch abscess)	7	6	1	0
DVT/PE	3	2	1	0
Wound dehiscence/Hematoma evacuation	3	3	0	0
Arthrofibrosis	30	1	2	27
Graft failure	35	1	0	34
**Total**	85	11	4	66

### Trainee Level and Surgical Time

**Figure 1:**
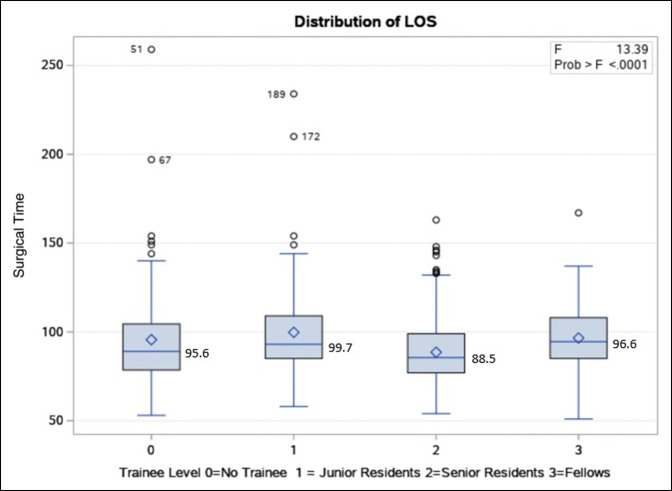
Graph showing the mean surgical time across trainee levels. Unadjusted bivariable analysis showing association between trainee level and mean surgical time.

Unadjusted bivariable analysis showed a significant difference in mean surgical time across trainee levels (*P* < 0.0001) (Figure 1), with senior residents having the shortest mean surgical time. The mean surgical time across trainee levels was 95.6 minutes with no trainees, 99.7 minutes with junior residents, 88.5 minutes with senior residents, and 96.6 minutes with fellows.

Across all aggregated cases, trainee level was significantly associated with increased surgical time (*P* = 0.0008), with procedures involving fellows having increased surgical times (*P* = 0.0011). Multivariable regression analysis with surgical time as the dependent variable showed that ACL reconstruction with concurrent meniscal repair or débridement (*P* < 0.0001), patient age (*P* < 0.0001), BMI (*P* = 0.0020), revision surgery (*P* < 0.0001), and attending surgeon (*P* < 0.0001) were statistically significant predictors of increased surgical time (Table 3). The effect sizes were calculated to be 0.1738 (procedure type), 0.0225 (age), 0.0124 (BMI), 0.0891 (revision surgery), and 0.1438 (attending surgeon).

**Table 3:** Multivariable Linear Regression Model of Factors Affecting Surgical Time

Surgical Time			
Parameter	Unadjusted β Coefficient (95% CI)	*P* value	Partial Eta Squared
Intercept	69.05 (61.1 to 77.0)	<0.0001	—
Sex (female)	−0.25 (−2.6 to 2.1)	0.8356	0.0001
Sex (male)	0	—	—
Age	−0.24 (−0.35 to −0.13)	<0.0001	0.0225
BMI	0.39 (0.14-0.63)	0.002	0.0124
Procedure type			0.1738
Isolated ACL repair	0	—	—
ACL repair, meniscal repair	18.14 (15.2-21.1)	<0.0001	—
ACL repair, meniscal débridement	7.59 (4.5-10.7)	<0.0001	—
ACL repair, chondroplasty/Loose	−0.38 (−6.2 to 5.5)	0.9037	
Body			0.0214
Trainee level			
No trainees	0	—	—
Junior residents	4.62 (−1.0 to 10.2)	0.1051	—
Senior residents	3.10 (−1.8 to 8.0)	0.2169	—
Fellow	8.93 (3.6-14.3)	0.0011	—
Revision surgery	18.70 (14.5-22.9)	<0.0001	0.0891
Total trainees	−0.94 (−4.2 to 2.3)	0.5655	0.0004
Attending surgeon			0.1438
1	38.20 (31.3-45.1)	<0.0001	—
2	10.0 (6.3-13.6)	<0.0001	—
3	3.23 (−0.1 to 6.6)	0.0607	—
4	11.70 (6.7-16.7)	<0.0001	—
5	0	—	—
History of diabetes	5.12 (−6.7 to 16.9)	0.3944	0.0009
Smoking status			0.0095
Current	−1.38 (−7.5 to 4.8)	0.6596	—
Former	10.50 (2.7-18.3)	0.0087	—
Never	0	—	—
R^2^ = 0.38			

ACL = anterior cruciate ligament, BMI = body mass index

### Trainee Level and Postoperative Complications

Short-term complications were rare in our cohort, with 1.9% of patients having complications less than 90 days after surgery. Trainee level was not an independent risk factor of short-term complications; the only notable risk factor of short-term complications was concurrent meniscal repair (OR = 4.6, 95% CI 1.3 to 16.3).

The overall global complication rate for our cohort was 10.6%, including longer term complications such as arthrofibrosis and graft failure. Trainee level was not a notable risk factor of global complications. Multivariable logistic regression modeling of risk factors of all postoperative complications did not show any significant risk factors (Table 4). Logistic regression models for each individual postoperative complication showed that trainee level was not a notable risk factor. Patient characteristics found to be notable risk factors of individual postoperative complications were age for DVT/PT, BMI for infection, and female sex for arthrofibrosis (Table 5).

**Table 4:** Logistic Regression Model of Risk Factors of Global Postoperative Complications

Risk Factor	Adjusted OR (95% CI)	*P* value
Sex	1.18 (0.75-1.88)	0.4687
Age	0.98 (0.96-1.00)	0.1598
Procedure type		
ACL repair + meniscal repair	1.79 (1.05-3.06)	0.0832
ACL repair + meniscal débridement	1.04 (0.55-1.96)	0.5706
ACL repair + chondroplasty	1.11 (0.27-4.51)	0.8828
Revision surgery	0.86 (0.37-2.04)	0.7344
Trainee level		
Junior residents	1.44 (0.48-4.31)	0.4437
Senior residents	1.57 (0.57-4.34)	0.1897
Fellow	0.79 (0.25-2.50)	0.1886
Total trainees	0.61 (0.28-1.33)	0.2115
Attending surgeon		
1	4.05 (1.25-13.17)	0.0700
2	1.98 (0.90-4.35)	0.9202
3	1.83 (0.87-3.84)	0.6626
4	2.30 (0.84-6.35)	0.6668

**Table 5:** Logistic Regression Model of Risk Factors of Individual Postoperative Complications

Complication	Risk Factor	Adjusted OR (95% CI)	*P* value
Infection	BMI	1.10 (1.00-1.20)	0.0387
DVT/PE	Age	1.07 (1.02-1.12)	0.0049
Wound dehiscence/Hematoma evacuation	BMI	0.837 (0.74-0.952)	0.0067
Arthrofibrosis	Sex (female vs. Male)	2.70 (1.27-5.74)	0.0096
Graft failure	Sex (female vs. Male)	0.42 (0.21-0.86)	0.0166
	Age	0.90 (0.84-0.96)	0.0014
	BMI	0.91 (0.83-0.99)	0.0358

BMI = body mass index

## Discussion

Trainee level is associated with surgical time, as surgeries took longer when fellows were involved, but not postoperative complications for ACL reconstructions. Differences in mean surgical time across trainee levels are likely attributable to different trainee levels operating with varying surgeons and participating in different procedure types. The lack of variance in surgical time between different levels of residents is consistent with a previous study on total knee arthroplasty.^6^ The factors that have a notable effect on mean surgical time included procedure type, revision surgeries, patient age, patient BMI, and attending surgeon. We found that procedure type and attending surgeon have the biggest effect on differences in surgical time. Differences in surgical time based on the attending surgeon could be attributed to the surgical technique, overall experience, case difficulty, and the relative amount of time spent teaching intraoperatively.

Operating as a surgical trainee is an essential step in the path to becoming an orthopaedic surgeon; however, few studies have assessed the effect of trainees on surgical time and postoperative complications in orthopaedics. Prior research has focused primarily on the effect of trainees on surgical time in nonorthopaedic specialties.^3-5^ However, studies have not explored how trainee level could affect surgical time or postoperative complications. Previous studies on factors affecting surgical time suggest that increased surgeon experience, team familiarity, and surgical volume could lead to shorter operating times.^12,13,14,15^

We also found that trainee level is not associated with an increased risk of postoperative complications. The most common short-term complications in our cohort included infection and wound dehiscence; neither of these complications was associated with trainee level or the number of trainees present in the procedure. These findings add to the growing body of evidence refuting the “July effect,” at least in the operating room. The July effect describes an increase in complications and infections when medical trainees transition between years.^16-18^ Trainee level was not a global risk factor of complications, nor for any of the individual complication types studied.

It is important to emphasize that this study was designed to assess the effect of trainee level on surgical time and short-term complications, rather than outcomes, and therefore, our findings are suggestive rather than definitive, particularly in light of an average follow-up of 283 days. Nevertheless, our findings that patient-specific risk factors of complications, such as increased BMI and infection risk^19^ or increased age and DVT risk,^20^ are supported by previous studies. We also found an increased risk of arthrofibrosis among women, confirming two previous studies.^21,22^ Our follow-up was admittedly short for assessing graft failure, which was associated with younger male patients with lower BMI in our current analysis. The association between increased age and decreased graft failure is consistent with previous studies.^23^ However, there is currently contradictory evidence regarding the effect of patient sex and BMI on the risk of ACL graft failure.^23,24,25,26^ More studies are needed to determine whether our findings hold up with longer follow-up or reflect the limitations and biases of our methodology.

## Limitations

Limitations of our investigation include study generalizability because we examined ACL reconstructions conducted at a single academic ASC. Most of our patients were young, healthy, and undergoing an elective procedure. As a result, our findings may not be broadly generalizable to patients with multiple comorbidities or to patients undergoing other surgeries. Most trainees in our study were senior residents (PGY4 or higher), which limited our ability to identify the effect that more junior trainees may have on surgical time or postoperative complications. We did not include other patient-specific and surgery-specific variables, such as graft choice, that could have affected surgical time. Additional research on factors that could affect the difference in surgical time, such as autonomy given to trainees, amount of intraoperative teaching, and surgeon experience, is needed.

## Conclusion

Our analysis of ACL reconstructions conducted in an academic ASC showed that resident trainee level is not markedly associated with increased surgical time or rates of postoperative complications. The presence of a fellow was associated with increased surgical time; however, other factors (including patient BMI, patient age, additional meniscal procedures, and attending surgeon) had a larger effect on surgical time. These findings suggest that this procedure is not negatively affected by medical education. Whether this holds up for other similar outpatient orthopaedic procedures in this setting is speculative and deserves investigation.
